# Study on Damage Mechanism of Fiber Concrete with Initial Pores

**DOI:** 10.3390/ma18050916

**Published:** 2025-02-20

**Authors:** Ankui Hu, Xinyu Du, Fei Wang, Junjie Li, Tianlong Zhang, Yajing Li

**Affiliations:** 1 School of Energy and Power Engineering, Xihua University, Chengdu 610039, China; dxy20010429@163.com (X.D.); feiwang201268@163.com (F.W.); junjie_li9038@163.com (J.L.); liyajing201808@163.com (Y.L.); 2 Key Laboratory of Fluid and Power Machinery of Ministry of Education, Xihua University, Chengdu 610039, China; 3 School of Civil Engineering and Architecture, Hainan University, Haikou 570228, China; ztl@hainanu.edu.cn

**Keywords:** finite element method, fiber-reinforced concrete constitutive, porosity, dynamic mechanical response

## Abstract

Currently, fiber-reinforced concrete, as a building material, is widely used in highway bridges and tunnel linings, and it has become a global research hotspot, with indoor tests, numerical simulations, performance studies, and application scenarios surrounding it. Many researchers have conducted experiments and analyses on the damage patterns of fiber-reinforced concrete under different conditions. However, there is relatively little research on the mechanical properties of fiber-reinforced concrete that already contains initial damage. This article establishes a micro-model composed of aggregates, mortar, and interface layers using MATLAB. It introduces the CDP (Concrete Damage Plasticity) constitutive equation for fiber-reinforced concrete and uses the least squares method to fit and validate the equation. After model validation, uniaxial compression tests are conducted on models with different initial porosities using the ABAQUS (2023) software, resulting in changes in crack damage, peak stress, and elastic modulus mechanical properties. The conclusions are as follows: The improved characteristic structure curve using the least squares method fits the experimental results well, and the rationality of the algorithm was verified by comparing it with physical tests. As the porosity increased from 2% to 8%, the peak stress decreased from 98.6% to 70.5% compared to non-porous fiber concrete with a significant rate of decrease of about 30%. After considering the strain rate, the peak stress increased slowly with increasing strain rate, but the elastic modulus increased at a significant rate, with a 1.26 times higher elastic modulus at a strain rate of 10^0^ than at a strain rate of 10^−2^. This result provides a certain theoretical basis for the mechanical properties and damage modes of fiber-containing concrete in practical engineering.

## 1. Introduction

Fiber concrete, characterized as a non-homogeneous porous building material comprising mortar, aggregate, an interface layer, fibers, and various other phases, is extensively utilized in infrastructure projects such as tunnel linings and water-retaining structures [1,2,3,4,5,6,7,8]. The quality of fiber concrete can be influenced by numerous factors, including construction techniques, vibration processes, and curing conditions. Curing conditions include ambient temperature and humidity [9]. The interplay of these factors can lead to the formation of defects, such as initial pores and cracks, within the concrete. While the incorporation of fibers is known to enhance the performance of concrete, the presence of initial damage alters the internal structure of fiber concrete, resulting in stress concentrated at the damage sites and promoting further crack propagation. Consequently, it is imperative to investigate the impact of initial damage on the mechanical properties of fiber concrete to ensure the safety and reliability of structural components in practical applications.

Currently, there are few studies on the damage mechanisms of fiber concrete, especially those containing different initial porosities. Concrete research on initial porosity focuses on experimental and numerical simulation. The experimental method is mainly used to measure the concrete pore parameters such as porosity, pore type and pore size distribution, etc., on the basis of which the link between the internal initial pore structure and the macroscopic properties of concrete, the form of damage, and durability can be further analyzed and investigated. Zingg [10] studied the relationship between the porosity, pore size distribution, and the compressive strength of concrete in different concrete specimens through experiments. Zhu et al. [11] investigated the effect of porosity on macroscopic compressive strength by analyzing the pore structure of concrete sections. Shi [12] investigated the effect of pore increment on the strength of C20 milled concrete and its size effect based on acoustic emission techniques. Zhao [13] et al. explored porousness as a cause of concrete compartmentalization through controlled tests under various loading schemes. Chen and other scholars [14] studied the pore structure of concrete by using the method of pores (MIP) and analyzed the compressive stress–strain response, predicted stress-strain response, and its relationship with the characteristics of the pore structure of concrete under high strain rates. Liu et al. [15] utilized computed tomography (CT) scanning to assess the impact of different aggregate particle sizes and cementitious material dosages on the internal pore structure of pervious concrete. Liu and colleagues [16] analyzed the destructive behavior of pore structures through acoustic emission phenomena. As for numerical simulation, Li et al. [17] used EPS (polystyrene foam) particles to simulate the pore structure in concrete, produced concrete specimens with different porosities, and measured relevant mechanical parameters through various tests. Xie [18] analyzed the crack evolution process and strain–strain curve of porous concrete under uniaxial compression using the finite element method. Nitka [19] et al. used numerical simulation to investigate the effect of initial microporosity on macroscopic and microscopic damage extension on the basis of considering the non-homogeneity of the concrete material. Wen [20] investigated the pore structure of fiber-reinforced antifreeze permeable concrete based on a hydrodynamic approach. Zhang et al. [21] studied the influence of initial porosity and aggregate gradation on concrete’s compressive and splitting tensile strengths, particularly in the context of EPS precast concrete. Similar investigations have been conducted through numerical simulations. Guo et al. [22] simulated the effects of varying numbers, sizes, and locations of initial pores on the mechanical properties of concrete by representing these pores as single or double entities.

Furthermore, for some kinetic application scenarios, studies on plain concrete are more common. It is essential to incorporate concrete structures into the design of critical civil and military protective facilities to ensure their resilience against explosions, impacts, and other significant dynamic loads, including seismic and explosive forces [23,24,25]. Research indicates that there are substantial differences in the mechanical properties of concrete when subjected to dynamic versus static loads. In their study, Ozbek et al. [26] examined the cracking behavior of porous concrete under impact loading and identified the primary factors influencing its dynamic mechanical properties. Additionally, Bai et al. [27] performed dynamic mechanical experiments on lightweight porous concrete utilizing a modified split Hopkinson compression bar.

A thorough examination of the aforementioned research indicates that the existing studies are mainly focused on the damage mechanism of ordinary concrete, while there are fewer studies on fiber concrete containing initial porosity, and the existing configuration for the internal preset initial pores is relatively simplistic, failing to adequately represent the randomness associated with the location, quantity, and size of pores in fiber-reinforced concrete. In addition, in the context of numerical methods, the establishment of a dynamic finite element model is often complex and requires extensive computational resources, which has led to a predominance of physical experiments in the investigation of the dynamic mechanical response of fiber-reinforced concrete. This paper establishes a model of fiber concrete with initial random porosity and verifies the rationality of the constitutive equations by comparing them with physical experiments. It explores a series of damage scenarios under uniaxial compression as well as the entire process of crack initiation, propagation, and penetration at different porosities. Additionally, it simulates the changes in mechanical properties of fiber-reinforced concrete under different strain rates.

## 2. The Establishment of Two-Dimensional Mesoscopic Model

### 2.1. Aggregate Grading Theory and Its Generation Criteria

To ensure that the generated fine view model of concrete closely resembles actual concrete, the generation algorithm for the fine view structure satisfies the following two criteria:

(1) It must accurately reflect the gradation of aggregates.
(2) The size, geometry, and positioning of the aggregates should be random and uniformly distributed, and the size and distribution of pores must also be random.

According to the shape and geometry of the model, meshing must be performed beforehand. Aggregates in concrete are of different sizes and are distributed in various grain size intervals. The distribution of aggregates determines the area and shape of the weak areas of stress within the concrete [28], and the stress distribution is affected by the distribution law of aggregates. W.B. Fuller believed that combining and matching the aggregates by a specific law of grain size produces concrete with smaller gaps and a higher density and put forward the ideal grading curve. Fuller’s ideal grading curve expression is shown in Equation (1) [29], which represents the spatial grading curve of aggregate in concrete.

(1) $$P=100\sqrt{\frac{D}{D_{\max}}}$$

where *P* (%) is the percentage of mass of the aggregates passing through the sieve diameter *D* (mm) and *D*_max_ (mm) is the maximum aggregate size.

Given that the optimal grading curve is formulated from a spatial three-dimensional model, which necessitates a substantial number of meshes and is computationally demanding, this study introduces a two-dimensional planar model. Walraven [30] proposed a formula based on the assumption of equal probability spatial distribution in order to ensure the equivalence of coarse aggregate gradation in a two-dimensional model. This formula can be seen in Equation (2), and it transforms the Fuller grading curve into a probability of diameter distribution within a two-dimensional cross-section.

(2) $$\begin{array}{l} P_{c}(D<D_{0})=P_{k}(1.065D_{0}^{0.5}D_{\max}^{-0.5}-0.053D_{0}^{4}D_{\max}^{-4}\\ -0.012D_{0}^{6}D_{\max}^{-6}-0.0045D_{0}^{8}D_{\max}^{-8}+0.0025D_{0}^{10}D_{\max}^{-10}) \end{array}$$

where *P*_c_ (%) is the probability of occurrence of different aggregate grades in the two-dimensional cross-section of the specimen. *D*_0_ (mm) is the particle size of the graded aggregate. *P_k_* (%) is the percentage of the aggregates in the total volume of concrete.

**Figure 1:**
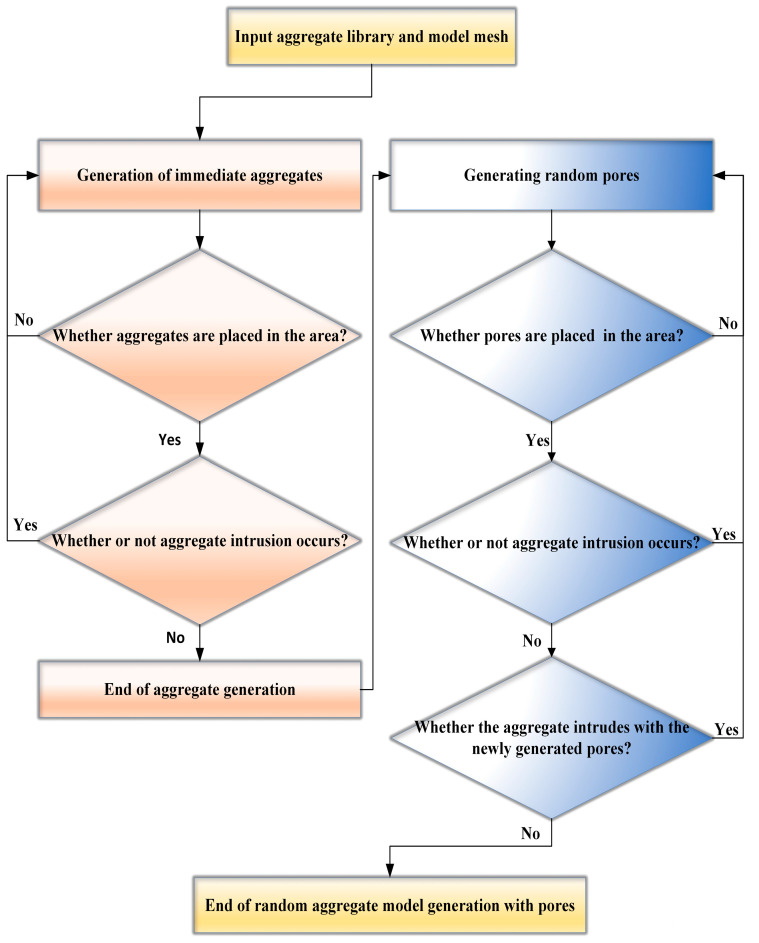
Flowchart for random aggregate and pore generation.

The steps for model concrete generation are as follows:

(1) Input the characteristic parameters of the model, including the sample size of the concrete, and proceed to mesh the specimen. Subsequently, determine the aggregates’ particle sizes and input the percentage of each particle size following the grading curve.
(2) The generation of random aggregates begins with the assignment of random locations for the aggregates, which are primarily governed by the coordinates of a randomly selected geometric center point. Following this, the random generation function in MATLAB is employed to produce a substantial number of random point coordinates within the designated simulation area. For each generated aggregate, it is essential to verify whether it overlaps with any previously generated aggregates or extends beyond the simulation boundaries. Overlap detection can be accomplished by calculating the distance between two spheres; if this distance is less than the sum of their respective radii, overlap is deemed to occur. For instance, if the coordinates of the centers of the spheres are (*x*_1_, *y*_1_, *z*_1_) and (*x*_2_, *y*_2_, *z*_2_) with radii *r*_1_ and *r*_2_, the overlap is confirmed if the following condition is satisfied.
  (3) $$\sqrt{(x_{1}-x_{2}{)}^{2}+{\left(y_{1}-y_{2}\right)}^{2}+{\left(z_{1}-z_{2}\right)}^{2}}<r_{1}+r_{2}$$
(3) Generation of random pores. Similar to the generation of the aggregates, random points are generated in the pore space, which are used as the intermediate positions of the pores. It is essential to assess whether these newly generated pores intersect with the previously established aggregates and other pores. This judgment is divided into the judgment of pore and aggregate and the judgment of intrusion between pores. In pore generation, the first step is to complete the contact judgement between the new pore and the old aggregate. If there is no contact, then continue to judge whether the newly generated pore and the old generated pore are in contact with each other. If not, the pore generation is successful. If the contact is found, then return to the initial step to re-generate the pore. The specific process is shown in Figure 1 below:

### 2.2. Fiber-Reinforced Concrete Constitutive Equation

In the context of the eigenstructure equation for fiber-reinforced concrete, it is common to approximate the CDP (Concrete Damage Plasticity) of conventional concrete as that of fiber-reinforced concrete, owing to the similarities observed in their stress–strain curves. However, this approximation frequently leads to significant discrepancies in the results. The eigenstructure employed in this study is derived from the damage eigenstructure equation for fiber-reinforced concrete, which was enhanced by Xu et al. [31], building upon the foundational principles of standard fiber-reinforced concrete.

One of the intrinsic model equations for compressive damage suggested in the Code for the Design of Concrete Structures (GB 50010-2010) [32] is as follows.

(4) $$\sigma =(1-d)\;\;E_{c}\varepsilon$$

(5) $$d=\left\{\begin{array}{ll} 1-\frac{\rho_{c}n}{n-1+x^{n}} & \left(x\leq 1\right)\\ 1-\frac{\rho c}{\alpha_{c}{\left(x-1\right)}^{2}+x} & \left(x>1\right) \end{array}\right.$$

(6) $$x=\frac{\varepsilon }{\varepsilon_{cu}}$$

(7) $$\rho_{c}=\frac{f_{c}}{E_{c}.\varepsilon_{cu}}$$

(8) $$n=\frac{1}{1-\rho_{c}}$$

where *σ* (MPa) is the stress; *ε* is the strain; *E_c_* (MPa) is the initial elastic modulus; *f*_c_ (MPa) is the representative value of uniaxial compressive strength; *ε_cu_* is the peak strain; *d* is the elastic damage variable; *x* is the normalized strain; *ρ_c_* is the corresponding stress degradation coefficient at the peak intensity; and *α_c_* is the control parameter for the descending section of the uniaxial compressive stress–strain curve.

Considering the effect of plastic deformation, the general expression for the elastic–plastic damage constitutive relationship [31] for concrete under uniaxial compression conditions is given by:

(9) $$\sigma =(1-D)E_{C}(\varepsilon -\varepsilon_{pl})$$

where *ε_pl_* is the plastic strain and *ε_ca_* is the additional strain. For the uniaxial compression unloading process, the unloading point is considered to be the current (*σ*, *ε*), so the following expression is obtained:

(10) $$\varepsilon_{pl}=\frac{d\varepsilon_{ca}}{(1-d)\varepsilon +\varepsilon_{ca}}\varepsilon$$

(11) $$\varepsilon_{\mathrm{ca}}=\frac{\varepsilon_{cu}}{\varepsilon_{cu}+\varepsilon }\sqrt{\varepsilon_{cu}\varepsilon }=\frac{\sqrt{x}}{1+x}\varepsilon_{cu}$$

(12) $$D=\frac{x(1+x)}{x(1+x)+\sqrt{x}}d$$

In this model, the main parameters are the modulus of elasticity *E_c_* (MPa), compressive strength *f_cu_* (MPa) and the corresponding strain *ε_c_*, plastic strain *ε_pl_*, and falling section curvature control parameter *f_c_* (MPa). A large number of test results show that the fiber has no significant effect on the peak strength, peak strain, or modulus of elasticity, while the falling section of the stress–strain curve of the concrete control parameter has a greater impact. After fitting, a large number of data can be obtained by the calculation of *α*_c_ using the expression [31]:

(13) $$\alpha_{c}=1.101+0.79e^{-3.308\lambda_{f}}$$

*α_c_* is related to the fiber characteristic parameter.

(14) $$\lambda_{f}=V\%\times l_{f}/d_{f}$$

The modulus of elasticity of concrete was adopted from the formula for the modulus of elasticity of ordinary concrete.

(15) $$E_{c(\mathrm{MPa})}=\frac{10^{2}}{2.2+\frac{34.7}{f_{cu,k}}}$$

where *l_f_* (mm) is the length of the fiber; *d_f_* (mm) is the diameter of the fiber; and *f_cu,k_* (MPa) is the standard value of concrete compressive strength.

According to the GB 50010-2010 code for the design of concrete structures [32] specification, the standard compressive strength of the standard specimen *f_cu_* = 40 MPa, the specimen’s modulus of elasticity, *E_c_* = 32,500 MPa. Can be obtained from the fiber concrete material’s stress, strain, and damage.

### 2.3. Material Parameters

The Interfacial Transition Zone (ITZ) is defined as a layer of porous, weakened mortar that adheres to the surface of the aggregates, demonstrating stable properties. The mechanical behavior of both the mortar and the ITZ is comparable to that of concrete, as indicated in the existing literature [33]. In consideration of computational costs and reference to established research models, the thickness of the ITZ is established at 1 mm, while its performance is enhanced to 70% of that of the mortar. To minimize the computational burden associated with numerical simulations, the model for the undamaged elastomer aggregate is utilized, with an elastic modulus of 70,000 MPa, a Poisson’s ratio of 0.2, and a density of 2690 kg/m^3^. The concrete employed in the model is designated as C40, exemplified by a cube model with a side length of 150 mm. The relevant details regarding the concrete are presented in Table 1.

### 2.4. Modeling

**Figure 2:**
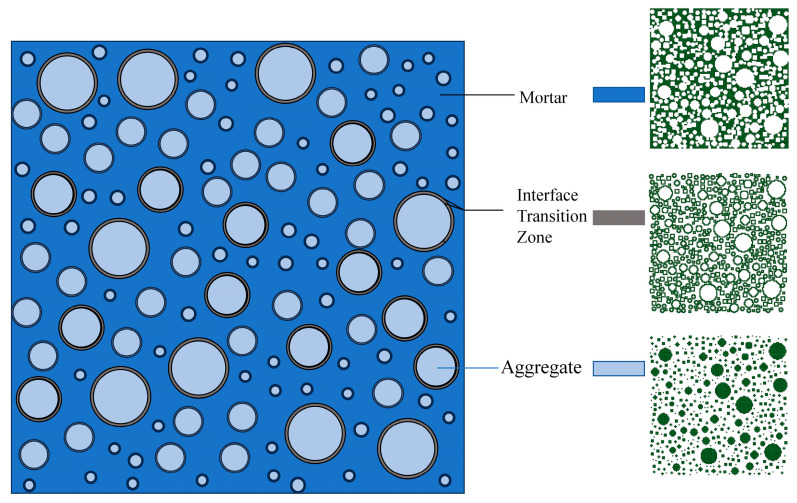
Parameters of the components of the microstructure of concrete.

In order to verify the reasonableness of the model, the above modeling method and the constitutive equations were used and simulated in ABAQUS, a large general-purpose finite element software, by comparing with the physical tests by Xu [31]. In this paper, the type of fiber selected was polypropylene fiber with a length of 8 mm, and the compressive strength value of the fiber concrete with an *L*/*D* ratio of 167 and a volume admixture of 0.05% was measured to be 33.4 MPa, where *L* refers to the length of the fiber and *D* refers to the diameter of the fiber. Fibers have a reinforcing effect on concrete, and its effect is reflected in the above structural equations. Thus, the fiber entities were not considered in the modeling process. The model is shown below in Figure 2. In this paper, the C3D4 solid unit was used for concrete, and the bonding effect between the two was not considered in the modeling process. In order to ensure the stability of the calculation results, displacement loading was used. The boundary conditions are fully constrained at the bottom of the model with a vertical downward displacement load applied at the top. Fiber concrete containing a 0.05% volume admixture, with a length of 8 mm and an *L*/*D* ratio of 167 input into the equation above, was simulated.

### 2.5. Finite Element Validation and Curve Fitting

**Figure 3:**
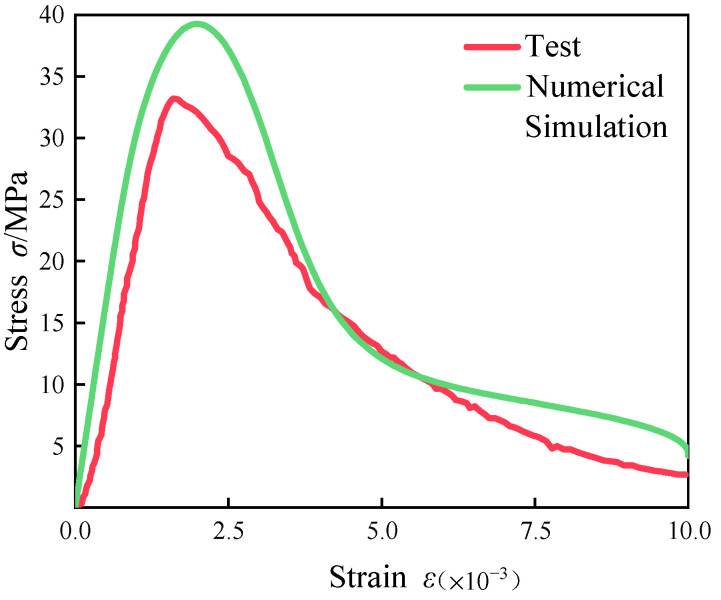
Comparison chart of constitutive curves and experimental curves.

We performed numerical simulations using the finite element method in the ABAQUS software. The stress–strain result obtained by bringing the above data into the intrinsic equation of fiber concrete was compared with the experimental result, as shown in Figure 3.

It is observed that the peak stresses obtained using the above constitutive equations were larger than the experimental values; of these, the error was about 18%, which was due to the presence of many influencing factors in the numerical simulations and physical experiments. Therefore, the constitutive equation needs to be corrected, which will in turn provide an important basis for subsequent comparison of the performance of fiber concrete containing initial defects. The fitting was carried out by adjusting the material stress ratio, where the adjustment ratio is *k*, and the corrected intrinsic equation is expressed as:

(16) $$\delta^{\prime }=k\times f(\varepsilon )$$

In this paper, the least squares method was used to determine the value of *k*. Since the difference between the simulated and experimental values was large at the peak stresses, the nearest multiple points were found. Suppose that at n feature point $\left(\delta_{i}^{sim},\varepsilon_{i}^{sim}\right)$(*i* = 1, 2, …, *n*), the corresponding simulation points are $\left(\delta_{i}^{\exp},\varepsilon_{i}^{\exp}\right)$ (*i* = 1, 2, …, *n*). Where exp represents the experimental value and sim represents the simulated value, the goal is to minimize the sum of squares of the errors between the corrected simulated curve and the experimental curve at these points. The error function is defined as:

(17) $$E(k)={\sum_{i=1}^{n}\left[k\times \delta_{i}^{\mathrm{sim}}-\delta_{i}^{\exp}\right]}^{2}$$

(18) $$E^{\prime }(k)=0$$

It is calculated that *k* equals 0.71. The modified constitutive equations were simulated numerically, and the fitted curves are shown below.

(19) $$\delta^{\prime }=0.71f(\varepsilon )$$

**Figure 4:**
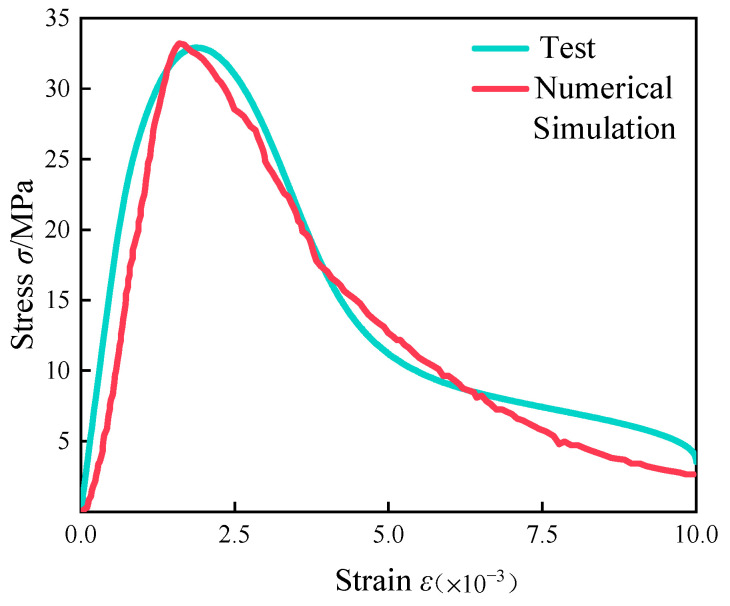
Comparison chart of the fitted curve and the experimental curve.

The stress–strain curve obtained from the modified constitutive equations was fitted to the experimental curve, as shown below in Figure 4.

From the above figure, it can be seen that the stress–strain curve obtained after using the above modeling method and the intrinsic equation of fiber concrete was in better agreement with the physical experiments. The error in peak stress between the numerical simulation and physical test was reduced from 18% to about 1%.

## 3. Simulation Results

### 3.1. Mechanical Properties of Fiber Concrete with Different Porosities

#### 3.1.1. Stress–Strain Curve

**Figure 5:**
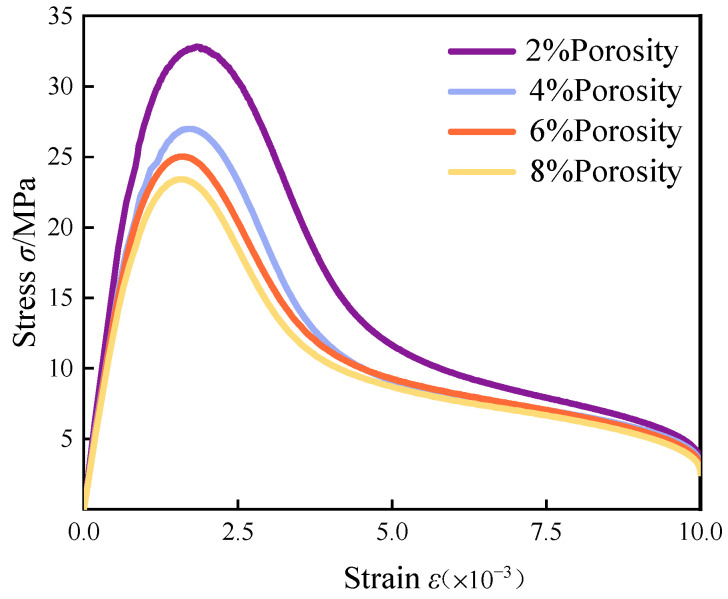
Comparison of uniaxial compression stress–strain curves with different porosities.

It is known through a large number of fiber concrete test data that the porosity of fiber concrete is generally in the range of 2 to 8% [34,35,36]; porosity here refers to the ratio of the size of the pore volume to the total. Using the method described in the previous section, we established models containing porosities of 2%, 4%, 6%, and 8%. The boundary conditions and loading methods were the same as in the previous chapter. The stress–strain curves at different porosities are shown below in Figure 5.

The analysis of the stress–strain curves reveals that as the porosity of fiber-reinforced concrete increased, the peak compressive stress progressively diminished. Specifically, for porosity levels ranging from 2% to 8%, the peak stress values were recorded at 98.6%, 81.3%, 75.4%, and 70.5% of the stress observed in non-porous concrete. At lower porosity levels, the mechanical properties of the concrete were less adversely affected; however, once porosity exceeded 4%, there was a marked decline in the strength of the fiber-reinforced concrete, with a maximum reduction approaching 30%. This finding underscores the significant influence of porosity on the peak uniaxial tensile stress of concrete.

In the initial phase of the stress–strain curve, the relationship appeared approximately linear, although the rate of increase in stress diminished as porosity rose. Concurrently, the initial modulus of elasticity experienced a gradual decline, and the peak of the curve became more rounded, lacking a pronounced steep incline or decline. Following the peak stress, an increase in porosity correlated with a downward trend in concrete strength. This phenomenon can be attributed to the fact that increased porosity compromises the density of the internal structure of the concrete, thereby reducing the contact area between the cement matrix and the aggregates. Consequently, the adhesive forces diminished under external loading, leading to stress concentrations around the pores, which heightened the likelihood of crack formation and propagation, ultimately resulting in a reduction in concrete strength.

#### 3.1.2. Displacement Distribution

**Figure 6:**
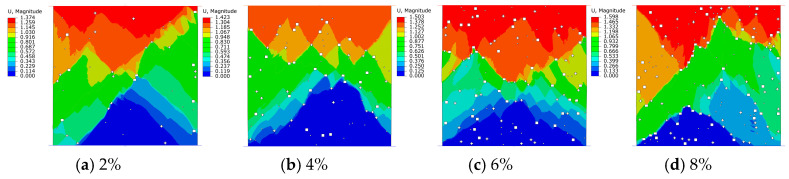
Comparison of displacements at different porosities.

In this paper, uniaxial compression simulation by the large-scale software ABAQUS was performed to calculate the damage stages of fiber concrete with different porosities, and four comparative cloud diagrams of (a~d) were output in the post-processing of the software. The displacement distribution of the fine-scale model with different porosities is shown in Figure 6. From the figure, it can be seen that in the vertical direction of the model (i.e., y-axis of the model), the overall displacement gradually decreased from along the loading direction, and the maximum displacement was distributed near the loading surface.

In addition, with the increase in porosity, the vertical displacement also increased from 1.374 mm at the time of destruction to 1.598 mm. The growth rate was as high as 16.3% during this period, which indicates that the level of porosity has a significant effect on displacement. The deformation of high porosity was larger than that of low porosity, which indicates that the stress concentration phenomenon inside the high porosity concrete accelerated the deformation of the internal structure of the concrete, and the larger deformation near the edges of the model and the pores during the loading process indicates that the model began to fracture at this point and gradually lost its load-bearing capacity.

#### 3.1.3. Macroscopic Crack Development Process

**Figure 7:**
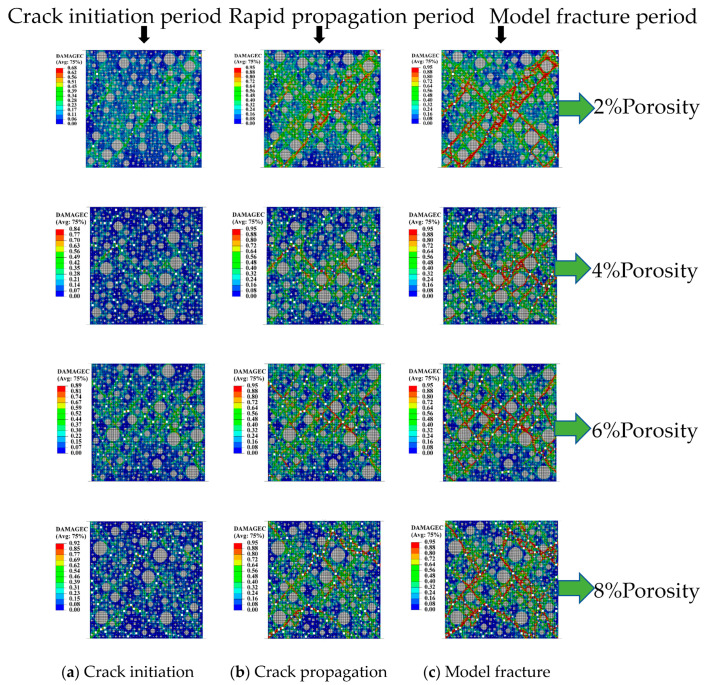
Crack damage evolution diagram for concrete with different pore fibers.

In order to further study the damage evolution process of fiber concrete with initial porosity under uniaxial compression, the crack expansion morphology of different fiber concretes at different porosities was observed at various stages, as shown in Figure 7.

Crack propagation can be categorized into three distinct stages based on observational analysis.

(1) The initial stage, known as crack initiation, as shown in column a, typically occurs at the material’s surface or at sites of internal stress concentration. During this phase, a small amount of unit damage occurred at localized locations of stress concentrations. Multiple microcracks were produced at the edges of several pores. Cracks tended to form around pores, and interface layers began to emerge.
(2) The subsequent stage is characterized as stable expansion, as shown in column b. During this period, the crack gradually developed to the main crack region, causing localized damage, and the unit damage was concentrated near the main crack region. The main crack sprouted a large number of small cracks, the number of damage units increased rapidly, and the length and width of the main crack region increased.
(3) The final stage, referred to as destabilized expansion, was marked by a rapid increase in crack length, leading to a swift and irreversible process that culminated in the formation of a macroscopic penetration crack, ultimately resulting in the specimen’s inability to sustain load. These observations align with the experimental results obtained in column c.

Furthermore, an examination of the damage evolution diagrams across varying porosities reveals the formation of numerous X-shaped cracks surrounding the pores. This phenomenon can be attributed to the stress concentration effects intensified by the presence of pores, which led to the earliest crack formations in these areas and subsequently facilitated the development of both large and small cracks along the pores. Notably, concrete samples with lower porosities, such as 2% and 4%, exhibited a limited number of cracks with a relatively uniform distribution area. In contrast, samples with porosities of 6% and 8% demonstrated a greater number of cracks across all three stages, with a wider and more concentrated distribution area. Additionally, the size of the pores significantly influenced crack distribution; smaller apertures tended to generate lower stress concentrations compared to larger ones. This observation is attributable to the relationship between the stress concentration factor and the shape and size of the pores. Specifically, smaller pore sizes resulted in a reduced area of interference with the overall structural stress distribution, thereby yielding a relatively lower stress concentration factor.

### 3.2. Uniaxial Compression Damage Analysis Under Different Loading Rates

#### 3.2.1. Peak Stress Analysis

The experimental study showed that the loading rate is an important factor affecting the macroscopic compressive strength of concrete with different humidities. In the solution process, ABAQUS calculated the change in node displacement to obtain the change in strain with time and then determine the strain rate. The displacements applied to the specimen were 0.15 mm, 1.5 mm, and 15 mm. Thus, it was dynamically loaded at different strain rates (10^−2^~10^0^) [37,38], and the specific cases are shown in Table 2.

**Figure 8:**
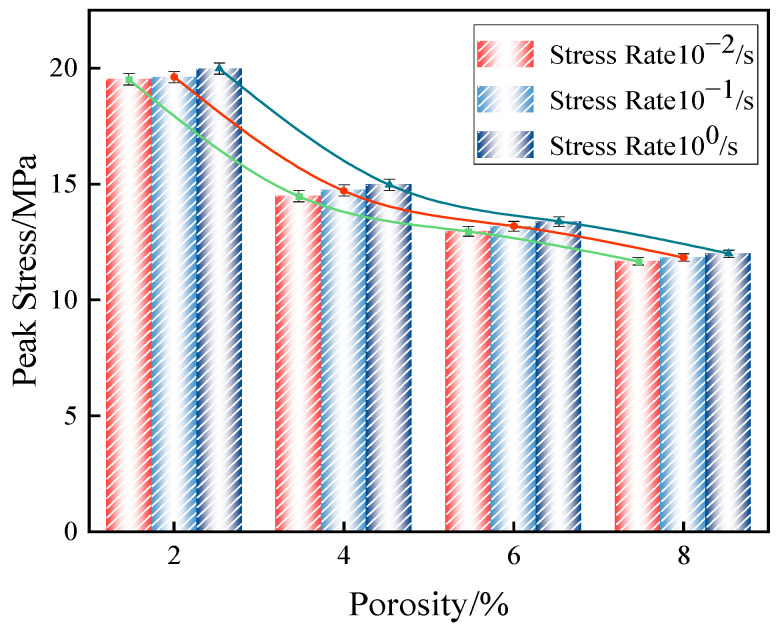
Graph of the relationship between the peak stress of fiber-reinforced concrete with initial porosity and strain rate.

Substituting the above 12 case into the ABAQUS software, the peak axial compressive stresses of concrete with initial pore fibers under different working conditions are shown in the table below. In order to show the data in Table 3 more clearly, the data were converged into Figure 8.

In comparison, it was observed that at a constant strain rate, an increase in porosity led to a more significant variation in the peak stress of fiber-reinforced concrete, exhibiting a linear relationship. Conversely, as the strain rate increased, the variation in peak stress was minimal, remaining within 1%. An internal analysis of the material indicates that, regardless of whether the strain was small or large, the weak regions within the concrete predominantly influenced the overall peak stress. This limited change in peak stress is attributed to the inherent properties of the material, which restricted the load-carrying capacity when only the loading rate was altered.

#### 3.2.2. Modulus of Elasticity Analysis

**Figure 9:**
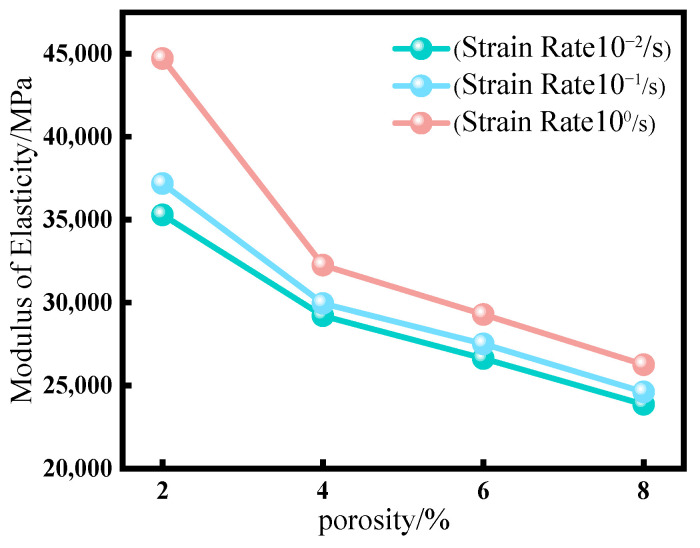
Graph of the relationship of the elastic modulus of fiber-reinforced concrete with initial porosity, porosity, and strain rate.

The modulus of elasticity mode selected in this paper is a linear interval of the uniform elastic deformation stage. The relationship between the uniaxial compressive modulus of elasticity and the porosity of concrete is shown in Figure 9. The specific data are shown in Table 4.

The data presented in the figure indicate that the modulus of elasticity of concrete subjected to uniaxial compression exhibited a gradual decline as porosity increased. Conversely, an increase in strain rate resulted in a significant rise in the modulus of elasticity, particularly at a strain rate of 10^0^, where it increased by a factor of 1.26 compared to a strain rate of 10^−2^. This phenomenon can be attributed to the material’s inertia during rapid loading, which impedes deformation and causes the material to resist changes in shape over a brief period, thereby resulting in a marked increase in the modulus of elasticity.

## 4. Discussion

Our research has discovered some changes in the mechanical properties of fiber-reinforced concrete with initial damage. The peak stress of fiber-reinforced concrete with initial pores decreased more significantly as the porosity increased. When the porosity reached 2%, the peak stress was 98.6% of that of non-porous concrete; when the porosity reached 8%, the peak stress dropped to 70.5% of that of non-porous concrete, with a nearly 30% decrease as the porosity increased stepwise. Additionally, when considering the strain rate, the dynamic compressive strength of fiber-reinforced concrete with initial pores increased with the rise in strain rate, although the growth trend was relatively slow. In contrast, the elastic modulus significantly increased with the strain rate, particularly noticeable at a strain rate of 10^0^, where it was 1.26 times that of the elastic modulus at a strain rate of 10^−2^.

Analyzing the reasons for this, when the porosity is low, there is still enough continuous matrix within the concrete to bear most of the stress, and the fibers can also play a role to some extent. However, as the porosity further increases, the pores begin to interconnect, forming larger defect areas. At this point, the phenomenon of stress concentration becomes more severe, with a large amount of stress concentrated around the pores, causing the material to fail under lower pressures, leading to a more pronounced decrease in compressive strength and elastic modulus. The presence of pores exacerbates the stress concentration around them, causing cracks to initiate at the pores and eventually leading to the formation of numerous cracks of various sizes along the pores. These phenomena can provide a certain reference for the understanding of crack evolution patterns in fiber-reinforced concrete in engineering practice.

Due to limitations in time and conditions, this paper did not conduct physical experiments on porous fiber-reinforced concrete to compare with the numerical simulation results, resulting in a lack of completeness. Fiber-reinforced concrete not only contains a certain amount of porosity but also has moisture inside. Therefore, future studies could combine both factors to preset pores and water molecules in real fiber-reinforced concrete to further simulate their mechanical properties.

## 5. Conclusions

This article considers the initial damage within concrete based on fiber-reinforced concrete. A mesoscopic model of concrete was established using the MATLAB software (R2024b), introducing the fiber-reinforced concrete CDP modified constitutive equation from ordinary concrete. Using this method, different porosity models were established in the ABAQUS software, and their mechanical properties were analyzed. This research can provide a theoretical basis for the mechanical performance of fiber-reinforced concrete in practical engineering applications such as tunnel linings and medium-sized highway bridges.

(1) After modifying the constitutive equation using the least squares method, the numerical simulation results showed significant improvement compared to previous results and were in good agreement with experimental data. The error in peak stress between the numerical simulation and physical test was reduced from 18% to about 1%. This result validates the rationality of the modified constitutive equation.
(2) The uniaxial compression calculation results of fiber-reinforced concrete with different porosities indicated that with the increase in porosity, the vertical displacement also increased from 1.374 mm at the time of destruction to 1.598 mm. The growth rate was as high as 16.3% during this period. The peak stress and elastic modulus of fiber-reinforced concrete with initial porosity gradually decreased as porosity increased. The peak stresses were 98.6%, 81.3%, 75.4%, and 70.5% for nonporous concrete. This characteristic was consistent with the performance of the fiber-reinforced concrete’s mechanical properties during construction and concrete production.
(3) In the damage evolution process of cracks, it was found that concrete with a high porosity exhibited a wider and greater number of crack distributions upon failure. In contrast, concrete with a low porosity had a more concentrated stress distribution and fewer cracks compared to high-porosity concrete. This result can provide a basis for structural safety in engineering, ensuring the long-term safety of buildings.
(4) After considering the combined effects of strain rate and porosity, it was found that the strain rate significantly affected the elastic modulus of fiber-reinforced concrete, particularly at a strain rate of 10^0^, where it increased by a factor of 1.26 compared to a strain rate of 10^−2^, while its impact on the reduction of concrete strength was not obvious. Therefore, for high strain rates such as in explosions, it is particularly important to consider the effect of strain rate on fiber-reinforced concrete.

## Figures and Tables

**Table 1:** Concrete plastic damage parameters.

	Density	Modulus of Elasticity	Poisson’s Ratio	Expansion Angle	Eccentricity	*f_b__o_*/*f_c__o_*	*K*	Coefficient of Viscosity
Unit	kg/m^3^	MPa	/	°	/	/	/	/
Aggregate	2690	70,000	0.2	30	0.1	1.16	0.6667	0.006
Interface Layer	2390	24,000	0.2					
Mortar	2390	30,000	0.2					

Note: *f_bo_* is the initial equivalent biaxial compressive strength. *f_c__o_* is the initial equivalent uniaxial compressive strength. *K* is used to describe the shape of the yield surface. Coefficient of viscosity considers the viscous effect of the material.

**Table 2:** Summary of models used for all cases.

Case	Porosity (%)	Strain Rate (s^−1^)
1	2%	10^−2^
2	4%	10^−2^
3	6%	10^−2^
4	8%	10^−2^
5	2%	10^−1^
6	4%	10^−1^
7	6%	10^−1^
8	8%	10^−1^
9	2%	10^0^
10	4%	10^0^
11	6%	10^0^
12	8%	10^0^

**Table 3:** Comparison of peak stress under different working conditions.

	Porosity	Stress Value/MPa			
Strain Rate/s^−1^		2%	4%	6%	8%
10^−2^		19.52	14.48	12.96	11.67
10^−1^		19.61	14.73	13.18	11.83
10^0^		19.98	14.97	13.38	11.99

**Table 4:** Comparison of elastic modulus under different working conditions.

	Porosity	Stress Value/MPa			
Strain Rate/s^−1^		2%	4%	6%	8%
10^−2^		35,288	29,205	26,633	23,870
10^−1^		37,172	29,955	27,520	24,614
10^0^		44,713	32,254	29,283	26,256

## Data Availability

The original contributions presented in this study are included in the article. Further inquiries can be directed to the corresponding author.

## References

[1] Mufti A.A., Bakht B., Banthia N., Benmokrane B., Desgagné G., Eden R., Erki M.A., Karbhari V., Kroman J., Lai D. (2007). New Canadian highway bridge Design code design provisions for fibre-reinforced structures. Can. J. Civil. Eng..

[2] Behfarnia K., Behravan A. (2014). Application of high performance polypropylene fibers in concrete lining of water tunnels. Mater. Des..

[3] Ghadban A.A., Wehbe N.I., Underberg M. (2018). Effect of Fiber Type and Dosage on Flexural Performance of Fiber-Reinforced Concrete for Highway Bridges. ACI Mater. J..

[4] Liu Y.W., Cho S.W. (2018). Study on application of fiber-reinforced concrete in sluice gates. Constr. Build. Mater..

[5] An D., Chen Z., Meng L.H., Cui G.Y. (2020). Application of fiber-reinforced concrete lining for fault-crossing tunnels in meizoseismal area to improving seismic performance. Adv. Mech. Eng..

[6] Li F.R., Chen G.X., Xu G.Z., Wu Y.Y. (2020). An Experimental Study on the Compressive Dynamic Performance of Polypropylene Fiber Reinforced Concrete for Retaining Structure under Automobile Collision Magnitude. Adv. Civ. Eng..

[7] Ryan C., Garcia-Taengua E. (2021). Fibre-Reinforced Concrete Is Sustainable and Cost-Effective for Water-Retaining Structures. Sustainability.

[8] Ruiz R., Todisco L., Corres H. (2023). Application of high-performance fibre reinforced concrete to precast girders for road bridges: Conceptual considerations and numerical analyses. Struct. Concr..

[9] Hájek M., Decký M., Walter S. (2016). Objectification of Modulus Elasticity of Foam Concrete Poroflow 17-5 on the Subbase Layer. Civ. Environ. Eng..

[10] Zingg L., Briffaut M., Baroth J., Malecot Y. (2016). Influence of cement matrix porosity on the triaxial behavior of concrete. Cem. Concr. Res..

[11] Zhu H.B., Yan M.Z., Li C. (2015). Analysis of the Influence of Porosity of Macroscopic Pore on Concrete Strength by Image Method. J. Build. Mater..

[12] Shi R. (2020). The Effect of Mechanical Properties of Rcc with Initial Pores. Master’s Thesis.

[13] Zhao C., Li Q.J., Zhong X.G., Zhang T.Y. (2021). Experimental study on basic mechanical properties of porous metal-concrete. Constr. Build. Mater..

[14] Chen X.D., Xu L.Y., Wu S.X. (2016). Influence of Pore Structure on Mechanical Behavior of Concrete under High Strain Rates. J. Mater. Civil. Eng..

[15] Liu G.Z., Hou Z.B., Liao L., Lu Y.B., Li Z.D., Wang Y.M. (2022). Analysis of Microscopic Pore Structure of Pervious Concrete. China Concr. Cem. Prod..

[16] Liu D.W., Zhang W.M., Jian Y.H., Tang Y., Cao K.P. (2024). Damage precursors of sulfate erosion concrete based on acoustic emission multifractal characteristics and b-value. Constr. Build. Mater..

[17] Li S.H., Liu H.Q. (2017). Coal Gangue Concrete Mechanical Properties Under Porosity Influence. Non Met. Mines.

[18] Xie C., Yuan L.J., Zhao M., Jia Y.H. (2020). Study on failure mechanism of porous concrete based on acoustic emission and discrete element method. Constr. Build. Mater..

[19] Nitka M., Tejchman J. (2020). Meso-mechanical modelling of damage in concrete using discrete element method with porous ITZs of defined width around aggregates. Eng. Fract. Mech..

[20] Wen F.S., Fan H.F., Zhai S.T., Zhang K.Q., Liu F.S. (2020). Pore characteristics analysis and numerical seepage simulation of antifreeze permeable concrete. Constr. Build. Mater..

[21] Zhang G.H., Wei X., Yang Z.D., Gu Y.S., Wang M.M. (2024). Influence of Mesoscopic Initial Pore Defects on Mechanical Properties of Concrete. J. Build. Mater..

[22] Guo Y., Wu X.T., Wang B.Z., Cheng C.Z., Feng X.K. (2023). Numerical simulation of dynamic compression of concrete with pores. J. Hefel Univ. Technol. Nat. Sci..

[23] Khodayari A., Sadeghnejad A., Azizinamini A. (2024). Numerical Investigation of a UHPC Connection Detail for Simple for Dead Load and Continuous for Live Load Steel Bridges in Seismic Areas. Constr. Mater..

[24] Liu J.Y., Li Y. (2023). Analysis of the explosion resistance of an I-shaped steel-concrete composite structure under contact explosion. Adv. Struct. Eng..

[25] Zha Z.X., Wang T., Wu K.W., Peng W., Wu Z.H. (2023). Experimental study on collapse modes of reinforced concrete wallboards under earthquake action. J. Asian Archit. Build..

[26] Ozbek A.S.A., Weerheijm J., Schlangen E., van Brengel K. (2013). Dynamic behavior of porous concretes under drop weight impact testing. Cem. Concr. Comp..

[27] Bai E.L., Xu J.Y., Lu S., Lin K.X., Zhang Y.M. (2018). Comparative study on the dynamic properties of lightweight porous concrete. RSC Adv..

[28] Baalbaki W., Benmokrane B., Chaallal O., Aietcin P.C. (1991). Influence of coarse aggregate on elastic properties of high-performance concrete. ACI Mater. J..

[29] Wittmann F.H., Roelfstra P.E., Sadouki H. (1985). Simulation and analysis of composite structures. Mater. Sci. Eng..

[30] Walraven J.C., Reinhardt H.W. (1981). Theory and Experiments on the Mechanical Behaviour of Cracks in Plain and Reinforced Concrete Subjected to Shear Loading. Heron.

[31] Xu L.H., Huang B., Li B., Chi Y., Li C.L., Shi Y.C. (2019). Study on the stress-strain relation of polypropylene fiber reinforced concrete under cyclic compression. China Civil. Eng. J..

[32] (2010). Code for Design of Concrete Structures.

[33] Zhou X.Q., Hao H. (2008). Mesoscale modelling of concrete tensile failure mechanism at high strain rates. Comput. Struct..

[34] Hu X.Y., Pang J.Y., Lei C.X. (2023). Study on pore structure and permeability of basalt fiber reinforced concrete under confining pressure. Compos. Sci. Eng..

[35] Zhao J., Li X.F., Guo L. (2023). Study on fractal dimension and mechanical properties of basalt polypropylene fiber concrete pore structure. Compos. Sci. Eng..

[36] Liu B., Li D., Fu Q., He L., Mai T.R. (2023). Applicability of fractal models for characterising pore structure of hybrid basalt-polypropylene fibre-reinforced concrete. Rev. Adv. Mater. Sci..

[37] Wu S.X., Chen X.D., Zhou J.K. (2012). Influence of strain rate and water content on mechanical behavior of dam concrete. Constr. Build. Mater..

[38] Zheng Q.Q., Hu H., Xu Y., Zhang T. (2022). Damage evolution models of static pre-loaded concrete under impact load based on the Weibull distribution. Case Stud. Constr. Mat..

